# Supplementary material for: Development of a plant-based dessert using araticum pulp and chickpea extract: Physicochemical, microbiological, antioxidant, and sensory characterization
Source: PLoS One. 2024 Aug 23;19(8):e0307640. doi: 10.1371/journal.pone.0307640 (PMC11343372; doi:10.1371/journal.pone.0307640)
Supplement: Attachment — Submitted filename: PONE-D-24-08819_reviewer AA EDIT 2.pdf [file pone.0307640.s001.pdf]

PLOS ONE

Development of a plant-based dessert using araticum pulp and chickpea extract: physicochemical, microbiological, antioxidant, and sensory characterization

--Manuscript Draft--

**Manuscript Number:**	PONE-D-24-08819
**Article Type:**	Research Article
**Full Title:**	Development of a plant-based dessert using araticum pulp and chickpea extract: physicochemical, microbiological, antioxidant, and sensory characterization
**Short Title:**	Development of a plant-based dessert using araticum pulp and chickpea extract
**Corresponding Author:**	Maria Carolina Mesquita<br>University of Brasilia: Universidade de Brasilia<br>Brasília, DF BRAZIL
**Keywords:**	araticum; chickpea; plant-based dessert; antioxidant activity; stability
**Abstract:**	The demand for plant-based products has increased in recent years, explained by several aspects, such as the inverse association between the consumption of plant-based products and the prevalence of chronic diseases, environmental consciousness, concern for sustainable food, and the increasing number of people adept to veganism and vegetarianism and/or with allergies to milk proteins. This study aimed to produce and characterize a plant-based dairy alternative dessert based on araticum pulp and chickpea extract without added sugar and fat.Three formulations were prepared: Formulation 1 (F1): 20% araticum pulp + 80% chickpea extract; Formulation 2 (F2): 30% araticum pulp + 70% chickpea extract; and Formulation 3 (F3): 40% araticum pulp + 60% chickpea extract. All formulations' centensimal composition, sensorial characteristics, viscosity, total phenolic content, antioxidant activity, and microbiological stability were analyzed during 28 days of storage at 4°C.The increase in the proportion of araticum pulp in the formulations reduced moisture (F1:84.2, F2:83.2, and F3:82.4), protein (F1:3.3, F2:3.2, and F3:3.0) content, and pH (F1:5.50, F2:5.18, and F3:4.91), and increased the TSS (F1:8.36, F2:8.98, and F310.63 º Brix), total phenolics content (F1: 4,677, F2: 6,943, and F3 10,112 gallic acid mmol/L) and antioxidant activity (F1: 1,974, F2: 3,664, and F3: 5.523). During the 28 days of storage at 4°C, the formulations F1 and F2 showed better stability of phenolic compounds and antioxidant activity; however, the formulation F3 showed acceptable microbiological quality up to 28 days of storage, higher viscosity (F1: 238.90, F2: 474.30, and F3:3,959.77 mPa.s), antioxidant capacity and better scores in sensory analysis.The present study showed that the plant-based dessert elaborated with araticum pulp and chickpea extract might be considered a potential dairy alternative product with high antioxidant activity, protein content, and a viscosity similar to yogurt; however, its sensory aspects need improvement.
**Order of Authors:**	Maria Carolina Mesquita
	Márcio Antônio Mendonça
	Raquel Braz Assunção Botelho
	Sandra Fernandes Arruda
	Eliana dos Santos Leandro
**Additional Information:**	
**Question**	**Response**
**Financial Disclosure**<br><br>Enter a financial disclosure statement that describes the sources of funding for the work included in this submission. Review the submission guidelines for detailed	The author(s) received no specific funding for this work.

requirements. View published research articles from *PLOS ONE* for specific examples.<br><br>This statement is required for submission and **will appear in the published article** if the submission is accepted. Please make sure it is accurate.<br><br>**Funded studies**<br>Enter a statement with the following details:<br>• Initials of the authors who received each award<br>• Grant numbers awarded to each author<br>• The full name of each funder<br>• URL of each funder website<br>• Did the sponsors or funders play any role in the study design, data collection and analysis, decision to publish, or preparation of the manuscript?<br><br>Did you receive funding for this work?	
**Competing Interests**<br><br>Use the instructions below to enter a competing interest statement for this submission. On behalf of all authors, disclose any competing interests that could be perceived to bias this work—acknowledging all financial support and any other relevant financial or non-financial competing interests.<br><br>This statement is required for submission and **will appear in the published article** if the submission is accepted. Please make sure it is accurate and that any funding sources listed in your Funding Information later in the submission form are also declared in your Financial Disclosure statement.<br><br>View published research articles from *PLOS ONE* for specific examples.	The authors have declared that no competing interests exist.

**NO authors have competing interests**<br><br>Enter: *The authors have declared that no competing interests exist.*<br><br>**Authors with competing interests**<br><br>Enter competing interest details beginning with this statement:<br><br>*I have read the journal's policy and the authors of this manuscript have the following competing interests: [insert competing interests here]*<br><br>* typeset	
**Ethics Statement**<br><br>Enter an ethics statement for this submission. This statement is required if the study involved:<br><br>• Human participants<br>• Human specimens or tissue<br>• Vertebrate animals or cephalopods<br>• Vertebrate embryos or tissues<br>• Field research<br><br>Write "N/A" if the submission does not require an ethics statement.<br><br>General guidance is provided below. Consult the submission guidelines for detailed instructions. **Make sure that all information entered here is included in the Methods section of the manuscript.**	This study is part of a larger research project that was approved by the Research Ethics Committee of the Faculty of Health Sciences at the University of Brasilia, Reference number nº 88754618.4.0000.0030/Emenda2/2023, dated March 31, 2023. The analysis took place at the Dietetic Technique Laboratory of the University of Brasilia, with 105 untrained participants recruited in a single day (May 31, 2023), who were randomly invited to participate in the research. They were instructed to read the Informed Consent Form, and the sensory analysis commenced only after their agreement and signature on the consent form (signed in writing). The inclusion criteria for participation in the research were being 18 years of age or older and not having an allergy and/or intolerance to chickpeas and araticum. The study did not include individuals under the age of 18.

Powered by Editorial Manager® and ProduXion Manager® from Aries Systems Corporation

**Format for specific study types**<br><br>**Human Subject Research (involving human participants and/or tissue)**<br>• Give the name of the institutional review board or ethics committee that approved the study<br>• Include the approval number and/or a statement indicating approval of this research<br>• Indicate the form of consent obtained (written/oral) or the reason that consent was not obtained (e.g. the data were analyzed anonymously)<br><br>**Animal Research (involving vertebrate animals, embryos or tissues)**<br>• Provide the name of the Institutional Animal Care and Use Committee (IACUC) or other relevant ethics board that reviewed the study protocol, and indicate whether they approved this research or granted a formal waiver of ethical approval<br>• Include an approval number if one was obtained<br>• If the study involved *non-human primates*, add *additional details* about animal welfare and steps taken to ameliorate suffering<br>• If anesthesia, euthanasia, or any kind of animal sacrifice is part of the study, include briefly which substances and/or methods were applied<br><br>**Field Research**<br><br>Include the following details if this study involves the collection of plant, animal, or other materials from a natural setting:<br>• Field permit number<br>• Name of the institution or relevant body that granted permission	
**Data Availability**<br><br>Authors are required to make all data underlying the findings described fully available, without restriction, and from the time of publication. PLOS allows rare exceptions to address legal and ethical concerns. See the PLOS Data Policy and FAQ for detailed information.	Yes - all data are fully available without restriction

Powered by Editorial Manager® and ProduXion Manager® from Aries Systems Corporation

A Data Availability Statement describing where the data can be found is required at submission. Your answers to this question constitute the Data Availability Statement and **will be published in the article**, if accepted.<br><br>**Important:** Stating 'data available on request from the author' is not sufficient. If your data are only available upon request, select 'No' for the first question and explain your exceptional situation in the text box.<br><br>Do the authors confirm that all data underlying the findings described in their manuscript are fully available without restriction?	
**Describe where the data may be found in full sentences. If you are copying our sample text, replace any instances of XXX with the appropriate details.**<br><br>• If the data are **held or will be held in a public repository**, include URLs, accession numbers or DOIs. If this information will only be available after acceptance, indicate this by ticking the box below. For example: *All XXX files are available from the XXX database (accession number(s) XXX, XXX.).*<br>• If the data are all contained **within the manuscript and/or Supporting Information files**, enter the following: *All relevant data are within the manuscript and its Supporting Information files.*<br>• If neither of these applies but you are able to provide **details of access elsewhere**, with or without limitations, please do so. For example:<br><br>*Data cannot be shared publicly because of [XXX]. Data are available from the XXX Institutional Data Access / Ethics Committee (contact via XXX) for researchers who meet the criteria for access to confidential data.*<br><br>*The data underlying the results presented in the study are available from (include the name of the third party*	All relevant data are within the manuscript and its Supporting Information files.

and contact information or URL).<br>• This text is appropriate if the data are owned by a third party and authors do not have permission to share the data.<br><br>* typeset	
Additional data availability information:	Tick here if the URLs/accession numbers/DOIs will be available only after acceptance of the manuscript for publication so that we can ensure their inclusion before publication.

Powered by Editorial Manager® and ProduXion Manager® from Aries Systems Corporation

Manuscript Click here to access/download;Manuscript;Manuscript.docx

Development of a plant-based dessert using araticum pulp and chickpea extract: physicochemical, microbiological, antioxidant, and sensory characterization

[1]Maria Carolina Mesquita, [3]Márcio Antônio Mendonça, [2]Raquel Braz Assunção Botelho, [2]Sandra Fernandes Arruda, [2]Eliana dos Santos Leandro

[1]Post-Graduate Program in Human Nutrition, Faculty of Health Sciences, Campus Universitário Darcy Ribeiro, University of Brasília, Brasília 70910-900, Brazil

[2]Department of Nutrition, Faculty of Health Sciences, Campus Universitário Darcy Ribeiro, University of Brasília, Brasília 70910-900, Brazil

[3]Faculty of Agronomy and Veterinary Medicine, University of Brasília, Brasília 70910-900, Brazil

[*] **Corresponding author**:

Maria Carolina Mesquita

Email: maria.carolinams@hotmail.com

Post-Graduate Program in Human Nutrition, Faculty of Health Sciences, Campus Universitário Darcy Ribeiro, University of Brasília, Brasília 70910-900, Brazil Phone: +55 (61) 3107 1634

Abstract

The demand for plant-based products has increased in recent years, explained by several aspects, such as the inverse association between the consumption of plant-based products and the prevalence of chronic diseases, environmental consciousness, concern for sustainable food, and the increasing number of people adept to veganism and vegetarianism and/or with allergies to milk proteins. This study aimed to produce and characterize a plant-based dairy alternative dessert based on araticum pulp and chickpea extract without added sugar and fat.Three formulations were prepared: Formulation 1 (F1): 20% araticum pulp + 80% chickpea extract; Formulation 2 (F2): 30% araticum pulp + 70% chickpea extract; and Formulation 3 (F3): 40% araticum pulp + 60% chickpea extract. All formulations' centensimal composition, sensorial characteristics, viscosity, total phenolic content, antioxidant activity, and microbiological stability were analyzed during 28 days of storage at 4°C.The increase in the proportion of araticum pulp in the formulations reduced moisture (F1:84.2, F2:83.2, and F3:82.4), protein (F1:3.3, F2:3.2, and F3:3.0) content, and pH (F1:5.50, F2:5.18, and F3:4.91), and increased the TSS (F1:8.36, F2:8.98, and F310.63 º Brix), total phenolics content (F1: 4,677, F2: 6,943, and F3 10,112 gallic acid µmol/L) and antioxidant activity (F1: 1,974, F2: 3,664, and F3: 5.523). During the 28 days of storage at 4°C, the formulations F1 and F2 showed better stability of phenolic compounds and antioxidant activity; however, the formulation F3 showed acceptable microbiological quality up to 28 days of storage, higher viscosity (F1: 238.90, F2: 474.30, and F3:3,959.77 mPa.s), antioxidant capacity and better scores in sensory analysis.The present study showed that the plant-based dessert elaborated with araticum pulp and chickpea extract might be considered a potential dairy alternative product with high antioxidant activity, protein content, and a viscosity similar to yogurt; however, its sensory aspects need improvement.

Keywords: araticum; chickpea; plant-based dessert; antioxidant activity; stability

1.Introduction

The demand for plant-based products has increased in recent years.There is an inverse association between the consumption of plant-based products and the prevalence of chronic diseases [1]. Also, environmental consciousness, concern for sustainable food systems, the increasing number of people adept to veganism and vegetarianism and/or with allergies to milk proteins (α-lactalbumin, β-lactoglobulin, and casein) and intolerance to lactose [2] are some of the reasons that explain the current growing demand for plant-based food products.

According to the Plant-Based Food Association, the sales of plant-based dairy alternatives (PBDAs) rose by 9% in 2018, while those from cow milk fell by 6%. In 2023, an increase of over 11% was expected for the overall market share of PBDA [3]. According to the EAT-Lancet Commission, the consumption of fruits, vegetables, nuts, and legumes will have to double, while animal-source foods (meat and dairy) must be reduced by more than 50% by 2050. Both actions are essential to improve health and bring environmental benefits [4].

Fruits have been identified as the basis for the preparation of plant-based dairy alternative products [5-7], as they are sources of vitamins, minerals, fibers, and also of natural compounds that provide flavor and aroma [8]. In this context, the Brazilian Cerrado fruits may constitute an alternative ingredient for plant-based dairy alternatives. In addition to their nutrient content, Cerrado fruits have a high content of bioactive compounds with high antioxidant capacity [9, 10]. Phenolic compounds such as

flavonoids, tannins, and anthocyanins represent the main bioactive compounds found in Cerrado fruits [11].

Among the fruits of the Brazilian Cerrado, araticum (*Annona crassiflora*) has unique sensory characteristics, attractive color, intense flavor, and exotic aroma, with high nutritional and technological potential. Araticum has a high content of phenolic compounds (728.17 mg GAE/100 g fresh weight) when compared to other fruits from the Cerrado, such as *araçá*, yellow mombin, *buriti*, *cagaita* (89.14, 98.97, 110.72 and 143.44 mg GAE/100 g fresh weight, respectively) [12]. In addition, araticum has a fibrous appearance, with 6.80 g of dietary fiber/100 g of pulp and some polysaccharides with prebiotic properties (1-kestose 3.00 mg, xylohexaose 1.47 mg, xylopentaose 0.94 mg, xylotriose 0.49 mg/g dry weight) [13, 14].

Although fruits are good sources of carbohydrates, fibers, micronutrients, and bioactive compounds, they are poor in protein and lipids compared to dairy products [15, 16]. Therefore, the protein content constitutes a challenge in developing plant-based dairy alternatives. In this context, chickpeas may be considered an adequate plant-based protein alternative for plant-based dairy alternatives. Chickpeas contain 18 to 29% protein, with essential amino acids such as lysine (0.96 g/100g), leucine (0.48 g/100g), phenylalanine (0.42 g/100g), isoleucine (0.36 g/100g) and valine (0.38g/100g). It is considered the third most cultivated legume in the world and presents low allergenicity [17, 18]. The use of chickpeas as a food ingredient is currently poorly explored in the literature. Its properties and interaction with other food matrices are poorly understood [18].

Considering that the araticum fruit is rich in nutrients and non-nutrients, such as dietary fiber and phenolic compounds, and that chickpea is a source of proteins, including essential amino acids, this study hypothesizes that the combination/interaction of these two products could result in a plant-based dessert alternative to dairy desserts. Thus, this

study aimed to produce and characterize a plant-based dairy alternative dessert based on araticum pulp and chickpea extract without added sugar and fat.

2. Materials and methods

2.1. Araticum pulp preparation

The araticum (*Annona crassiflora*) fruits were purchased from a local street market in Brasília, Federal District, Brazil, and harvested from February to March 2022. The fruits were sanitized, cut crosswise, and the carpels removed. The seeds were removed from the pulp, and the pulp was packed in plastic bags under vacuum and stored at – 80°C.

2.2. Chickpea extract preparation

The chickpeas were purchased at a local market in Brasília, Federal District, Brazil (Bella Grão, Cristalina-GO, Brazil). Chickpea extract was prepared according to the process described by Rincon *et al*. [19].

2.3. Formulations preparation

In order to determine the best percentage of araticum pulp and chickpea extract to produce a plant-based dessert alternative well-accepted by consumers, three formulations with different proportions of araticum pulp and chickpea extract were prepared: Formulation 1 (F1): 20% araticum pulp + 80% chickpea extract; Formulation 2 (F2): 30% araticum pulp + 70% chickpea extract; Formulation 3 (F3): 40% araticum pulp + 60% chickpea extract.

To prepare the formulations, araticum pulp and chickpea extract were homogenized in a blender (Philips Walita, model ProBlend 4) for 30 seconds at power

one and then 30 seconds at power two. The homogeneous mixture was strained in voile to obtain the dessert. The preparation process of chickpea extract, araticum pulp, and formulations is summarized in a flowchart (Figure 1).

Aliquots of 400 mL of each formulation were placed in glass jars with screw caps and pasteurized at 65ºC for 15 minutes in a water bath (Warmnest, model HH-S10). After pasteurization, samples were cooled and stored at 4°C for 28 days.

2.4. Determination of centesimal composition and energy value

The moisture content, protein, lipid, mineral residue, crude fiber, and total carbohydrates of formulations were analyzed. Furthermore, the energy value of the formulations was estimated using the Atwater general factor system that includes energy values of 4 kcal per gram (kcal/g) for protein, 4 kcal/g for carbohydrates, and 9 kcal/g for lipid. Moisture was determined according to the Analytical Procedures of Instituto Adolfo Lutz [20]. Protein content was determined according to the Kjeldahl 991.22 method [21]. The lipid content was determined according to the Am 5-04 extraction method [22]. Mineral residue was determined using method 945.45 [21]. Crude fiber content was determined according to method 978.10 [21]. The total carbohydrate amount was determined by difference, subtracting the values found for moisture, protein, lipid, and crude fiber from 100, according to the method 986.25 [21]. All analyses were assayed in triplicate.

2.5. Stability of formulations during storage times

The physical-chemical and microbiological parameters of the different formulations, F1, F2, and F3, were analyzed on days 1, 7, 14, 21, and 28 of storage at 4°C.

2.5.1. pH and Total Soluble Solids (TSS)

The pH of the formulations was determined using a bench pHmeter (Digimed®, model DM21), using 10 g of sample, according to the Analytical Procedures of the Instituto Adolfo Lutz [20].

Total Soluble Solids (TSS) were determined using a digital refractometer (ATAGO®, model 1 T), according to the method 932.12 [21], and the results were expressed in ° Brix. All samples were assayed in triplicate.

2.5.2. Microbiological analyzes

The presence of mesophilic aerobic microorganisms, molds, and yeasts in the different formulations was analyzed according to the methodology described by the American Public Health Association [23]. Initially, the formulations were subjected to serial dilutions (10^{-1}, 10^{-2}, and 10^{-3}) in 0.85% (w/v) saline solution. The mesophilic aerobe microorganisms were determined by plating them in a standard agar culture medium (Neorgen®, Lansing, Michigan, USA). The plates were incubated in a bacteriological oven at 37°C for 48 hours, and the number of Colony Forming Units (CFU) was determined. The population of molds and yeasts was determined by plating them in a DRBC agar culture medium (Neorgen®, Lansing, Michigan, USA). The plates were incubated in a bacteriological oven at 25°C for five days, and the number of Colony Forming Units (CFU) was determined. Results were expressed in Colony Forming Units (CFU)/mL formulation. All samples were assayed in triplicate.

2.5.3. Determination of total phenolic content and antioxidant activity

The antioxidant activity and the total phenolic content of raw material used to elaborate the formulations, araticum pulp, chickpea extract, and the formulations (F1, F2, and F3) were evaluated. All the samples were analyzed before and after the pasteurization

process on days 1, 7, 14, 21, and 28 of storage at 4 ºC. All samples were assayed in triplicate.

2.5.3.1. Sample preparation

Sample extracts were prepared according to the protocol described by Larrauri *et al.* [24], with some modifications. Aliquots of 0.25 g of each sample were placed in microtubes, 2 mL of methanol/water (50:50 v/v) were added, and the reaction system remained under stirring for 1 hour at room temperature. Afterward, samples were centrifuged at 25,400 *xg* for 15 minutes at 25ºC, and the supernatant was collected. To the pellet, 2 mL of acetone/water (70:30 v/v) was added, and the previous procedure was performed. The methanol and acetone extracts were combined, and the final volume was adjusted to 5 mL with Mili-Q water to determine antioxidant activity and total phenolic content.

2.5.3.2. Determination of total phenolic

The determination of total phenolics was carried out according to the protocol described by Singleton and Rossi [25], which consists of the ability of phenolic compounds to reduce the mixture of Phosphomolybdic/Phosphotungstic acids in an alkaline medium. The determination of total phenolics was carried out in a 96-well plate, and the absorbance was measured at 765 nm, after 60 minutes of reaction under protection from light, in a spectrofluorimeter (SpectraMax M Series Multi-Mode Microplate Readers, San Jose, California). A standard curve of gallic acid was constructed at concentrations from 0 to 1,000 µmol/L, with a linear range of R^2 0.99. The results were expressed as µmol gallic acid/L of the formulation, araticum pulp, or chickpea extract.

2.5.3.3. Antioxidant activity

The antioxidant activity of the samples was evaluated by assays of free radical scavenging capacity (2,2-diphenyl-1-picrylhydrazyl (DPPH$^{\bullet}$) and ferric reducing antioxidant potential (FRAP).

The ability to scavenge free radicals was determined using the radical 2,2-diphenyl-1-picryhydrazyl (DPPH$^{\bullet}$), according to the protocol described by Cheng *et al.* [26]. The analysis was performed in a 96-well plate, and the absorbance was monitored at 515 nm every 30 seconds for 40 minutes in a spectrofluorimeter (SpectraMax M Series Multi-Mode Microplate Readers, San Jose, California). The antioxidant potential of the samples was calculated according to Equation 1. The results were expressed as mol TROLOX/L of the formulation, araticum pulp, or chickpea extract.

$$\text{DRSC}= \frac{AUC\ Sample}{AUC\ TROLOX}\, x\, \frac{[Sample\ \mu\text{L}]}{[TROLOX\ \mu\text{L}]} \qquad (1)$$

Where: DSRC is the relative DPPH$^{\bullet}$ radical scavenging capacity; AUC is the area under the curve plotted from the variables “sample volume” x “% DPPH$^{\bullet}$ scavenged”.

The FRAP assay was performed according to the protocol described by Benzie and Strain [27]. The FRAP reagent was prepared using 0.3 mol/L acetate buffer (pH 3.6), 10 mmol/L 2,4,6-triazinetripyridyl in 40 mmol/L hydrochloric acid, and an aqueous solution of 20 mmol/L ferric chloride in a proportion of10:1:1, and incubated at 37 °C for 30 min. The assay was performed by mixing 180 μL of FRAP reagent, 6 μL of sample extract, and 18 μL of deionized water. The analysis was performed in a 96-well plate, and the absorbance was measured at 593 nm, for 10 minutes of reaction in a spectrofluorimeter (SpectraMax M Series Multi-Mode Microplate Readers, San Jose, California). A standard curve was constructed with Trolox in a range of 0 – 1,500 μmol /L. The results were expressed as µmol TROLOX/L of the formulation, araticum pulp, or chickpea extract.

2.5.4. Determination of viscosity

To verify whether a higher proportion of araticum pulp could confer greater viscosity to the final product, the same proportions of araticum pulp used in the different formulations were homogenized in water and used as a control for the experiment: Control 1 (C1): 20% araticum pulp + 80% water; Control 2 (C2): 30% araticum pulp + 70% water; Control 3 (C3): 40% araticum pulp + 60% water. A natural whole yogurt (Itambé®, natural milk) was used as a viscosity standard.

The viscosity of the formulations and their controls was determined using a digital rotational viscometer Brookfield, model MVD-8 (Marte científica, Santa Rita do Sapucaí, MG, Brazil). Aliquots containing 10 g of each sample at room temperature (25 ºC) were used. The spindles #21, #22 and #23, were applied for 30 seconds, and the speed was set to 3, 6, or 12 rpm. The viscosity of the samples was analyzed at two storage times, days 1 and 28 at 4 ºC, and the results were expressed in mPa-s. All samples were assayed in triplicate [28, 29].

2.5. Sensory analysis

Sensory analysis was conducted according to the guidelines established in the Declaration of Helsinki and approved by the Research Ethics Committee of the University of Brasília (nº 88754618.4.0000.0030/Emenda2/2023). The analysis took place at the Dietetic Technique Laboratory of the University of Brasilia, with 105 untrained participants recruited in a single day (May 31, 2023), who were randomly invited to participate in the research. Participants were instructed to read the Free and Informed Consent Form, and the sensory analysis started only after agreement and signature of the term. The inclusion criteria were: being 18 years or older and not having any allergy and/or intolerance to chickpeas and araticum.

Formulations F1, F2, and F3, described in item 2.3, were prepared and pasteurized one day before the sensorial analysis and submitted for evaluation by the participants. About 25 g of the formulations were placed in plastic cups and coded with three digits. Each participant received an empty cup to discard the formulations, a glass of water to drink between evaluations, a napkin, and disposable spoons. The formulations were presented randomly to the participants. They evaluated color, taste, odor, texture, and overall acceptance through a 9-point hedonic scale, 9 corresponding to “I liked extremely” and 1 “I disliked extremely” [30].

2.6. Statistical analysis

All analyses were performed in triplicate. Data were expressed as mean ± standard deviation. Analysis of variance (ANOVA) was performed with subsequent Tukey’s test, considering $P < 0.05$ for significant differences. A regression analysis was done for variables analyzed in the storage time intervals, such as pH and TSS. SAS software (SAS Institute Inc., Cary NC, version 9.4) was used for data analysis, and SigmaPlot software (Systat Software, Inc., Palo Alto, CA, version 14.0) was used to plot the graphs.

3. Results and discussion

3.1. Centesimal composition

The centesimal composition of the different formulations is described in Table 1. As expected, the increase in the proportion of araticum pulp in the formulations resulted in a reduction in moisture (84.26 - 82.38 g/100 g) and protein content (3.38 - 3.04 g/100 g). These results are in agreement since chickpea extract has higher average moisture (93.7 ± 1.5 g/100g) and protein (2.1 ± 0.07 g/100g) concentration [19] compared to araticum pulp (73.08 ± 0.05 and 1.37 ± 0.05 g/100g, respectively) [31].

It is worth noting that the protein content observed in the formulations was similar to that obtained for yogurt and higher than other vegetable desserts based on soy, rice, coconut, macadamia, pea protein, and fava beans [32, 33]. The higher carbohydrate content in araticum pulp (18.92 ± 0.05 g/100g) [31] resulted in an increase of this nutrient in F2 (10.72 ± 0.05) and F3 (11.06 ± 0.06) in relation F1 (9.68 ± 0.15 g/100g) formulation. However, no difference was observed in carbohydrate concentration between F3 and F2 formulations (10.72 ± 0.05 and 11.06 ± 0.06 g/100g, respectively), despite the higher percentage of araticum pulp.

A similar result was observed for crude fiber content. The F3 showed a higher content of this nutrient (1.34 ± 0.05 g/100g) than F1 and F2 (0.86 ± 0.00 and 0.93 ± 0.11 g/100g, respectively). No difference was observed in crude fiber content between F1 and F2, despite the higher percentage of araticum pulp. The fiber content of the formulations was similar to other desserts made from almonds, cashews, oats, macadamia, and rice [33].

Regarding energy value, similar results were obtained for the formulations, with values lower than 200 kcal/100 g. No significant differences ($P > 0.05$) were obtained for lipid and ash concentration among all formulations with the increase in the proportion of araticum pulp. Araticum pulp and chickpeas extract have low lipid content (2.39 ± 0.05 and 0.39 ± 0.22 g/100g, respectively), which may explain these results. The low calorie and lipid content of the formulations can be considered positive factors, as consumers seek plant-based dairy alternatives that are creamy and healthy, without excessive calories and fats [33].

3.2. Stability during storage times

3.2.1. pH and TSS

The pH of the formulations, with different proportions of araticum pulp and chickpea extract, during 28 days of storage at 4 ºC are presented in Figure 2 and Table 2. Araticum is considered an acidic fruit, with a pH around 4.0 [34, 35], therefore as the proportion of araticum pulp increased in the formulations (F1: 20%, F2: 30% and F3: 40% araticum pulp to chickpea extract, respectively), the average pH values decreased (5.50 ± 0.03, 5.18 ± 0.07 and 4.91 ± 0.09 for F1, F2 and F3, respectively), regardless of storage time. At day 7 of storage at 4ºC, all formulations showed a significant decrease ($P < 0.05$) in pH values (5.55, 5.29, and 5.04 on day1; 5.50, 5.15, and 4.85 on day 7, for F1, F2, and F3, respectively; Fig. 2). Formulation F1 maintained the pH value from day 7 to day 28 of storage. However, the formulations F2 and F3 presented a decrease from day 1 to 7, an increase from day 7 to 14, and a reduction in pH values from day 14 to 21.

The decrease in the pH values may be related to the degradation of components, such as polyphenols, during storage and the rapid conversion of proteins to amino acids, resulting in greater acidity [36]. Some studies have reported similar behavior for pH values in products developed with fruits [36-38]. The formulation F3 showed the lowest pH values ($P < 0.05$) during the storage time compared to F1 and F2 formulations. The decrease in pH values in formulations can be considered a positive aspect since a lower pH value can inhibit or reduce the growth of pathogenic microorganisms [36].

Concerning the total soluble solids (TSS), there was a significant reduction ($P < 0.05$) in ºBrix values until day 14 of storage at 4ºC, regardless of the formulation composition, while no difference was observed in TSS values from day 14 of storage onwards (Fig 3 A and Table 2). Regardless of the storage time, the increase of araticum pulp in formulations resulted in significantly ($P < 0.05$) higher TSS medium values (8.36 ± 0.02, 8.98 ± 0.31, and 10.63 ± 0.39 º Brix for F1, F2, and F3 formulations, respectively; Fig. 3 B).

TSS is a group of substances that can be dissolved in water, including sugar. The decrease in TSS may be related to chemical reactions during storage, making them less soluble [39, 40]. Some studies have reported similar behavior for TSS in fruit products [37, 41]. The increase in araticum pulp in the formulations promoted an increase in TSS, and the formulations F2 and F3 showed a significantly higher TSS ($P < 0.05$) when compared to F1. Araticum pulp has a lower moisture content (73%) [31] than chickpea extract (93%) [19], which may explain the increment in total soluble solids as its proportion in formulation increases. Therefore, the lower moisture content in F2 and F3 formulations resulted in higher TSS.

3.2.2. Microbiological analysis

The count of mesophilic aerobic microorganisms, molds, and yeasts in the three formulations at days 1, 7, 14, 21, and 28 of storage at 4 ºC is described in Table 3. The F3 formulation, which has the higher proportion of araticum pulp, showed a low count of mesophilic aerobic microorganisms, molds, and yeasts (< 10 CFU/mL) during all 28 days of storage at 4 ºC. The F1 and F2 formulations presented a similar profile concerning mesophilic aerobic microorganisms, molds, and yeast count. From day 14 to day 28 of storage at 4 ºC, both formulations showed exponential growth of all studied microorganisms (F1: 4.3×10^2 to 1.3×10^6; F2: 1.4×10^2 to 4.2×10^5 CFU/mL for mesophilic aerobic count and F1: 3.2×10^2 to 1.6×10^5; F2: 3.1×10^2 to 1.2×10^5 CFU/mL for molds and yeasts).

Mesophilic aerobic, molds and yeasts are microorganisms that indicate the microbiological quality of food. Formulations F1 and F2 presented acceptable results for up to 21 days of storage at 4 ºC, as counts greater than 10^4 of the microorganisms analyzed are considered unsatisfactory with unacceptable quality [42, 43]. However, only the F3 formulation achieved acceptable quality results during 28 days of storage at 4 ºC. It should

be emphasized that none of the formulations were added of food additives to prevent the growth of microorganisms and thus extend the shelf life of desserts.

The low growth of these microorganisms in the formulations, especially in F3, may be related to the decrease in pH obtained by adding araticum pulp in the formulations. It is important to highlight that even though the pH of the three formulations is close to the ideal pH for the growth of molds and yeasts, 4 - 6, a low count of mesophilic aerobic microorganisms, molds, and yeasts was obtained, suggesting that some compounds in araticum pulp have antimicrobial activity against the studied microorganisms [44].

Plants from the *Annonaceae* family, such as araticum, have secondary metabolites, such as alkaloids, acetogenins, and phenolic compounds with antibacterial activity [45]. Da Silva *et al*. [46], detected alkaloids in araticum pulp and attributed the antimicrobial activity against S. aureus ATCC 6538 to these compounds. Ramos *et al.* [47], when analyzing the araticum fruit, detected acetogenins, such as anonacinone and squamolinone, in the pulp and cited that these compounds presented antibacterial activity. Stafussa *et al*. [48], identified some phenolic compounds, such as quinic acid and catechin, in araticum pulp and attributed the antibacterial activity of araticum pulp against *S. aureus* and *E. coli* to these compounds. Therefore, the higher proportion of araticum pulp and antimicrobial compounds in the F3 formulation may explain the lower growth of microorganisms compared to the other formulations.

The antimicrobial activity of the compounds cited above is associated with the hydroxyl groups present in their chemical structures, which may destabilize bacterial cell walls and/or inhibit bacterial enzymes, delaying the growth of microorganisms [49].

3.2.3. Total phenolic content

The total phenolic content of araticum pulp, chickpea extract, and different formulations during storage at 4°C for 28 days is shown in Table 4. Araticum pulp had higher total phenolic content (22,796 ± 729 µmol gallic acid/L) compared to chickpea extract (1,456 ± 713 µmol gallic acid/L, Table 4). As expected, the total phenolic content of the three formulations increased proportionally to the concentration of araticum pulp . The F3 formulation presented the higher phenolic content (10,112 ± 250 gallic acid µmol/L) compared to F1 and F2 formulations (F1: 4,677 ± 365 and F2: 6,943 ± 474 gallic acid µmol/L, respectively). These values were higher than those obtained for dairy and soy milk desserts, 1,970 to 4,560 gallic acid µmol/L [50], which demonstrates the potential of formulations made with araticum pulp and chickpea extract.

Vasco, Ruales & Kamal-Eldin [51], when categorizing fruits according to their phenolic content, suggested that those fruits with a phenolic content greater than 500 mg GAE/100 g fresh weight could be considered fruits with high levels of these compounds. Therefore, araticum pulp could be considered a fruit source of phenolic compounds. Arruda *et al.* [49], identified 139 phytochemicals in araticum pulp, of which 82 were phenolic compounds and their derivatives, representing 59% of all the identified phytochemicals. The authors also showed that 54% of the identified phenolic compounds were flavonoids. Some identified phenolic compounds were procyanidin, catechin, epicatechin, flavanomaein, quecetin, kaempferol, rutin, and caffeic acid. In addition to their antioxidant and antibacterial activities, the phenolic compounds have antitumor, anti-inflammatory, antihypertensive, and hepatoprotective properties [35, 52].

In the chickpea extract, the values of total phenolic content remained constant during the storage time of 28 days at 4°C. On day 7 of storage at 4°C, the phenolic content in the three formulations decreased compared to day 1. In the F1 and F2, no difference

was observed from day 7 to day 28 of storage in total phenolic content; however, in the formulation F3 (araticum pulp 40%), a significant decrease in phenolic content was observed on day 21 of storage compared to day 7.

These results suggest that the formulations with lower percentages of araticum pulp and consequently higher percentages of chickpea extract, F1 and F2, showed better stability of phenolic compounds during the 28 days of storage at 4ºC. The better stability of phenolic compounds in F1 and F2 formulations concerning F3 may be explained by the higher concentration of proteins, as phenolics interact with proteins. According to Guan *et al.* [53], phenolic compounds can interact with the peptide chains of vegetable and/or animal proteins through covalent and non-covalent bonds, and the conjugated complexes exhibit better stability, which preserves the antioxidant activity.

GopikaJayaprakash *et al.* [54], suggested that the interaction between rice protein and polyphenolic components of kidney beans increase vegan proteins' quality and commercial acceptability. Türkyılmaz *et al.* [55], reported that the amino acids aspartic acid, proline, and valine increased anthocyanins stability in a pomegranate and orange juice blend. Therefore, the presence of these amino acids in chickpea protein [56] reinforces the hypotheses that interactions between chickpea proteins and/or amino acids and the phenolic compounds of araticum pulp may have improved the stability of phenolic components during the storage of formulations with a higher percentage of chickpea extract.

The lower stability of total phenolics in F3 formulation throughout storage at 4ºC may be related to the decrease in the pH value during storage, which may diminish the stability of phenolic compounds. According to Ozdal Capanoglu and Filiz Altay [57], one of the main parameters that can affect the phenol-protein interaction is pH, as high values or values below neutrality (< 7) interfere with the degree of bonding between molecules.

A pH closer to the protein and/or amino acid's isoelectric point would be ideal for a stronger bond.

3.2.4. Antioxidant activity ($DPPH^{\bullet}$ and FRAP)

The antioxidant activity of araticum pulp, chickpea extract, and different formulations during storage at 4ºC for 28 days is shown in Table 4.

Araticum pulp showed higher antioxidant potential in both $DPPH^{\bullet}$ and FRAP assays compared to chickpea extract (64.6 ± 11.7 *versus* 3.1 ± 0.4 mol/L and 18,970.0 ± 363.9 *versus* 1,467.8 ± 39.5 µmol/L; Table 4). The antioxidant activity of araticum pulp significantly decreased from day 1 to 7 of storage at 4ºC, showing stability from day 7 of storage onwards. No significant differences were observed in the antioxidant potential of chickpea extract during storage time. The greater antioxidant activity in araticum pulp can be attributed to its higher total phenolic content when compared to chickpea extract, as mentioned previously.

The similarity between the results regarding total phenolic content and antioxidant capacity can be explained by the biological antioxidant activity exerted by phenolic species. Thus, a food's antioxidant potential can be attributed mainly to its total phenolic content [53].

The antioxidant activity of the different formulations estimated by the FRAP assay increased proportionally to the added content of araticum pulp. However, according to the $DPPH^{\bullet}$ assay, the antioxidant activity of the formulations F1 and F2 was not significantly different ($P > 0.05$). During the 28 days of storage, the antioxidant capacity of F1 and F2 formulations remained constant, which reinforces the hypothesis of a possible interaction between phenolic compounds of araticum pulp and proteins from chickpea extract, which results in a better stability of the antioxidant activity of the

formulations throughout storage at 4 ºC. Contrary to that observed for F1 and F2, the formulation F3, 40% araticum pulp + 60% chickpea extract, showed an increase in antioxidant potential from day 1 to 7 by the FRAP assay and a decrease by the DPPH$^{\bullet}$ assay, followed by stability in values from day 7 to 28 of storage, by both assays.

The difference in the antioxidant capacity measured by the FRAP and DPPH$^{\bullet}$ methods can be explained by the differences in the reactivity of various antioxidants in the chemical reaction of each method. FRAP method is based on the ability to reduce ferric tripyridyltriazine complex (Fe (III)-TPTZ) to blue ferrous complex (Fe (II)-TPTZ) by electron-donating antioxidants transfer, therefore measuring the reduction of the ferric ion. DPPH$^{\bullet}$ method is based on the scavenging of the 1,1-diphenyl-2-picrylhydrazyl free radical and, therefore, it measures the radical neutralization capacity of antioxidants [58].

In the present study, the antioxidant capacity measured by the FRAP method seems to be correlated with the total phenolic content of formulations, and both are proportional to the percentage of added araticum pulp to the formulations. Thaipong *et al.* [59], reported that the antioxidant activity estimated by FRAP assay had a higher correlation with phenolic content than that obtained using other assays such as DPPH$^{\bullet}$, ABTS$^{\bullet,}$ and ORAC.

In general, when observing the results of antioxidant capacity measured by the FRAP, the different formulations exhibit superior results compared to those obtained for dairy and soy milk desserts, ranging from 640 to 1,290 µmol/L [50]. Although formulation F3 showed lower stability in the first seven days of storage at 4ºC, its higher total phenolic content and greater antioxidant activity demonstrate the functional potential of the araticum and chickpea extract vegetarian dessert. It is well known that antioxidants can neutralize free radicals/oxygen reactive species, preventing various chronic diseases and consequently promoting health [58, 60].

3.2.5. Viscosity

The viscosity of the different formulations and respective controls are presented in Figure 4. Formulation 3 (40% araticum pulp + 60% chickpea extract) presented the highest viscosity (3,959.77 mPa.s) with F1 (238.90 mPa.s) and F2 (474.30 mPa.s). A similar result was obtained for its control (40% araticum pulp + 60% water). Although the F2 formulation has 1.5-fold more araticum pulp than the F1 formulation, no difference was observed in viscosity between these formulations (474.30 and 238.90 mPa.s, respectively). These results agree with that obtained for their respective controls; C2's (30% araticum pulp + 60% water) viscosity was similar to C1's (20% araticum pulp + 60% water). Regardless of the percentage of araticum pulp, the viscosity of formulations did not alter after 28 days of storage ($P > 0.05$).

Although the addition of 40% araticum pulp to 60% water increased the viscosity of the mixture (1,307.43 mPa.s) compared to 20% araticum pulp + 80% water (171,87 mPa.s), the combination of araticum pulp with chickpea extract in the same proportion, 40% araticum pulp to 60% chickpea extract, showed a 3-fold increase (3,959.77 mPa.s) compared to the control, 40% araticum pulp + 60% water (1,307.43 mPa.s). This result suggests an interaction between the components of the araticum pulp and chickpea extract matrices.

The higher viscosity value obtained for F3 may be related to the higher concentration of constituents with hydroxyl groups derived from araticum pulp in this formulation. Hydroxyl groups increase the affinity of the compounds to water, acting as natural hydrocolloids, resulting in increased viscosity [61].

In addition, some polysaccharides and proteins are considered hydrocolloids; however, most hydrocolloids are fibers [61, 62]. The F3 formulation had a higher fiber

content among formulations, and among the polysaccharides already identified in araticum pulp [35, 63]. Pectin is a hydrocolloid that provides greater firmness in foods [64]. It has also been reported that chickpea proteins can form gels [65]. The proportion of 40% araticum pulp and 60% chickpea extract in F3 was possibly sufficient for an interaction of the components, providing greater viscosity.

It is important to highlight that the viscosity of the F3 formulation was similar to that obtained for a commercial yogurt (3,233.80 mPa.s), used as a standard in the analysis, and no additives were added to increase the viscosity of the formulations. This result suggests that formulation F3 can be considered a plant-based dairy alternative.

3.3. Sensory analysis

The results of the sensory evaluation of the different formulations are described in Table 5. One hundred four tasters participated in the study, of which 68% were women and 32% were men, 80% were between 18-24 years and 20% were between 25 -54 years.

The best hedonic classification for color and taste was obtained for formulation F3. The odor score was similar among all formulations, while the texture attribute was similar between F2 and F3 formulations and higher for these two formulations than formulation F1. Despite the high values obtained for color and taste attributes in F3 formulation, the overall sensory score of F2 and F3 formulations did not differ; however, it was significantly higher than F1 formulation.

In general, hedonic classification varying between the terms "disliked moderately" to "indifferent" was observed for the different aspects evaluated in the formulations, with an exception for the color aspect of the F3 formulation, which remained within the acceptance scale "liked moderately". For a product to have good acceptability, it is advised to achieve around 70% acceptance in sensory parameters;

values below this percentage were obtained for the formulations. The low scores obtained in sensory evaluation may be associated with the striking flavor of araticum pulp, as it is considered an exotic fruit from the Brazilian Cerrado [13, 34]. Schiassi *et al.* [12], obtained similar results when conducting sensory analysis of fruit juices from the Brazilian Cerrado, and the authors suggested that the flavors of these unconventional fruits are not familiar to tasters, leading to low test scores.

Araticum pulp has a yellow/pink pigment [66], therefore the higher proportion of araticum pulp in the formulation F3 intensified its color, making it more attractive to tasters, which explains the greater acceptance for the color aspect in this formulation.

Despite the low hedonic classification of the formulations in the present study, these results were similar to those obtained for other plant-based desserts made with soy, rice, cocoa butter, cashew, coconut, and blueberry, available on the market [67-69]. Rincon *et al.* [19], demonstrated that the addition of natural flavorings, such as vanilla extract, can be a strategy to enhance the acceptability of plant-based milk. It is possible that such strategies could be applied to plant-based desserts to improve acceptability.

However, the formulation F3 presents the highest score in the sensorial aspects and appears to be the most promising formulation for a new plant-based dessert alternative.

4. Conclusion

The present study showed that the plant-based dessert elaborated with araticum pulp and chickpea extract might be considered a potential dairy alternative product, as it has a protein content and viscosity similar to yogurt. Furthermore, araticum pulp and

chickpea extract plant-based dessert may be a functional food, as it has a high content of phenolic compounds and, consequently, antioxidant activity.

The developed formulation with 40% araticum pulp and 60% chickpea extract (F3) presented the best potential as a plant-based dairy alternative dessert, as it had a viscosity similar to yogurt, acceptable microbiological quality up to 28 days of storage, and the highest antioxidant potential.

Despite the good nutritional value of the araticum pulp and chickpea extract plant-based dessert, the low sensorial acceptability of this dessert suggested that more studies are needed to refine the sensory aspects of these plant-based desserts.

Conflict of Interest

The authors confirm that they have no conflicts of interest with respect to the work described in this manuscript.

CRediT authorship contribution statement

Maria Carolina Mesquita: Conceptualization, Methodology, Investigation, Data Curation, Visualization, Writing - Original Draft, Project Administration. **Márcio Antônio Mendonça:** Resources, Data Curation. **Raquel Braz Assunção Botelho:** Resources, Data Curation, Writing - Review & Editing. **Sandra Fernandes Arruda:** Conceptualization, Validation, Resources, Methodology, Visualization, Writing - Review & Editing. **Eliana dos Santos Leandro:** Conceptualization, Methodology, Resources, Writing - Original Draft, Supervision.

Acknowledgment

We thank Coordenação de Aperfeiçoamento de Pessoal de Nível Superior (CAPES) of Brazil for the support with the scholarship, as well as the financial backing provided by the Fundação de Amparo a Pesquisa do Distrito Federal (FAPDF) – Process: 00193-00000104/2019-86.

5. References

1. Springmann M, Godfray HCJ, Rayner M, Scarborough P. Analysis and valuation of the health and climate change cobenefits of dietary change. Proc Natl Acad Sci. 2016; 113:4146–51.

2. Plamada D, Teleky BE, Nemes SA, et al. Plant-Based Dairy Alternatives—A Future Direction to the Milky Way. Foods. 2023; 12(9): 1–33. https://doi.org/10.3390/foods12091883

3. Mintel Group Ltda. Milk and Non-Dairy Milk—US. Available online: https://reports.mintel.com/display/919346/. Accessed 14 Jan 2024.

4. Springmann M, Wiebe K, Mason-D'Croz D, Sulser TB, Rayner M, Scarborough P. Health and nutritional aspects of sustainable diet strategies and their association with environmental impacts: a global modelling analysis with country-level detail. Lancet Planet Health. 2018; 2: e451–61.

5. Mulwa SN, Mahungu SM, Muinde BK. Effect of Soursop Puree and Gum Arabic on the Sensory Properties of Non-Dairy Coconut. Food Nutr Sci. 2023; 14(7): 670–86. https://doi.org/10.4236/fns.2023.147044

6. Pimentel TC, Gomes de Oliveira LI, Carvalho de Souza R, Magnani M. Probiotic non-dairy frozen dessert: Technological and sensory aspects and industrial challenges. Trends Food Sci Technol. 2021; 107(October 2020): 381–88. https://doi.org/10.1016/j.tifs.2020.11.008

7. Bocker R, Silva EK. Innovative technologies for manufacturing plant-based non-dairy alternative milk and their impact on nutritional, sensory and safety aspects. Future Foods. 2022; 5(July 2021): 100098.

8. Rodríguez LG, Zamora Gasga VM, Pescuma M, Van Nieuwenhove C, Mozzi F, Sánchez Burgos JA. Fruits and fruit by-products as sources of bioactive compounds. Benefits and trends of lactic acid fermentation in the development of novel fruit-based functional beverages. Food Res Int. 2021; 140(July 2020). https://doi.org/10.1016/j.foodres.2020.109854

9. De Almeida AB, Silva AKC, Lodete AR, Egea MB, Lima MCPM, Silva FG. Assessment of chemical and bioactive properties of native fruits from the Brazilian Cerrado. Nutr Food Sci. 2019; 49(3): 381–92. https://doi.org/10.1108/NFS-07-2018-0199

10. Siqueira EMA, Rosa FR, Fustinoni AM, de Sant'Ana LP, Arruda SF. Brazilian

savanna fruits contain higher bioactive compounds content and higher antioxidant activity relative to the conventional red delicious apple. PLoS One. 2013; 8(8): e72826. https://doi.org/10.1371/journal.pone.0072826

11. Bailão EFLC, Devilla IA, da Conceição EC, Borges LL. Bioactive compounds found in Brazilian cerrado fruits. Int J Mol Sci. 2015; 16(10): 23760–83. https://doi.org/10.3390/ijms161023760

12. Schiassi MCEV, Souza VR de, Lago AMT, Campos LG, Queiroz F. Fruits from the Brazilian Cerrado region: Physico-chemical characterization, bioactive compounds, antioxidant activities, and sensory evaluation. Food Chem. 2018; 245(October 2017): 305–11. https://doi.org/10.1016/j.foodchem.2017.10.104

13. Arruda HS, Pereira GA, Pastore GM. Oligosaccharide profile in Brazilian Cerrado fruit araticum (*Annona crassiflora* Mart.). LWT - Food Sci Technol. 2017;76:278–83. https://doi.org/10.1016/j.lwt.2016.05.017

14. Arruda HS, Pereira GA, Pastore GM. Brazilian Cerrado fruit araticum (*Annona crassiflora* Mart.) as a potential source of natural antioxidant compounds. Int Food Res J. 2018; 25(5): 2005–12.

15. Vincente AR, Manganaris GA, Ortiz CM, Sozzi GO, Crisosto CH. Nutritional Quality of Fruits and Vegetables. In: Postharvest Handling: A Systems Approach; 2014. p. 69–122. https://doi.org/10.1016/B978-0-12-408137-6.00005-3

16. Lagrange V, Whitsett D, Burris C. Global market for dairy proteins. J Food Sci. 2015; 80(S1): A16–22. https://doi.org/10.1111/1750-3841.12801

17. Rachwa-Rosiak D, Nebesny E, Budryn G. Chickpeas—Composition, Nutritional Value, Health Benefits, Application to Bread and Snacks: A Review. Crit Rev Food Sci Nutr. 2015; 55(8): 1135–43. https://doi.org/10.1080/10408398.2012.687418

18. Boukid F. Chickpea (*Cicer arietinum* L.) protein as a prospective plant-based ingredient: a review. Int J Food Sci Technol. 2021; 56(11): 5435–44. https://doi.org/10.1111/ijfs.15046

19. Rincon L, Botelho RBA, de Alencar ER. Development of novel plant-based milk based on chickpea and coconut. LWT - Food Sci Technol. 2020; 109479.

20. IAL (Instituto Adolfo Lutz). Métodos físico-químicos para análise de alimentos. 4th ed. São Paulo: Instituto Adolfo Lutz; 2008.

21. AOAC (Association of Official Analytical Chemists). Official Methods of Analysis of AOAC International. 18th ed. AOAC International; 2005.

22. AOCS (American Oil Chemists' Society). Approved Procedure Am 5-04 Rapid Determination of Oil/Fat Utilizing High Temperature Solvent Extraction. http://www.academia.edu/30938058/AOCS. Accessed 25 Mar 2023.

23. APHA (American Public Health Association). Compendium of Methods for the Microbiological Examination of Foods. 4th ed. Downes & Ito; 2001.

24. Larrauri JA, Rupérez P, Saura-Calixto F. Effect of Drying Temperature on the Stability of Polyphenols and Antioxidant Activity of Red Grape Pomace Peels. J Agric Food Chem. 1997; 45(4): 1390–3. https://doi.org/10.1021/jf960282f

25. Singleton VL, Rossi JA. Colorimetry of total phenolics with phosphomolybdic-

phosphotungstic acid reagents. Am J Enol Vitic. 1965; 16(3): 144–58.

26. Cheng Z, Moore J, Yu L. High-throughput relative DPPH radical scavenging capacity assay. J Agric Food Chem. 2006; 54(20): 7429–7436. https://doi.org/10.1021/jf0611668

27. Benzie IFF, STrain JJ. The ferric reducing ability of plasma (FRAP) as a measure of "antioxidant power": The FRAP assay. Anal Biochem. 1996; 239(292): 70–6. https://doi.org/10.1039/c6ay01739h

28. Damian C, Oroian MA, Leahu A, Cioarbă I. Effect of addition of starch and agar-agar on rheological behaviour of yogurt. Food Environ Saf J. 2012; 11(1).

28. Gheshlaghi SP, Khaledabad MA, Nikoo M, Regenstein JM, Gavlighi HA. Impact of sturgeon gelatin hydrolysates (SGH) on physicochemical and microbiological properties of fat-free set-type yogurt. LWT - Food Sci Technol. 2021; 148: 111665. https://doi.org/10.1016/j.lwt.2021.111665

30. Stone H, Bleibaum R, Thomas HA. Sensory evaluation practices. 4th ed. New York: Academic Press; 2012. p. 81–115.

31. Midlej LC, Arruda SF, Mesquita MC, Mendonça MA, Leandro E dos Santos. Araticum (*Annona crassiflora*) juice is an excellent matrix for Lactobacillus paracasei LBC-81 with high antioxidant potential. J Funct Foods. 2023; 111(November). https://doi.org/10.1016/j.jff.2023.105883

32. Aryana KJ, Olson DW. A 100-Year Review: Yogurt and other cultured dairy products. J Dairy Sci. 2017; 100(12): 9987–10013. https://doi.org/10.3168/jds.2017-12981

33. Craig WJ, Brothers CJ. Nutritional Content of Non-Dairy Frozen Desserts. Nutrients. 2022;14(19): 1–10. https://doi.org/10.3390/nu14194150

34. Cardoso L de M, Oliveira D da S, Bedetti S de F, Martino HS D, Pinheiro-Sant'ana HM. Araticum (*Annona crassiflora* Mart.) from the Brazilian Cerrado: Chemical composition and bioactive compounds. Fruits. 2013; 68(2): 121–34. https://doi.org/10.1051/fruits/2013058

35. Arruda HS, Pastore GM. Araticum (*Annona crassiflora* Mart.) as a source of nutrients and bioactive compounds for food and non-food purposes: A comprehensive review. Food Res Int. 2019; 123(March): 450–80. https://doi.org/10.1016/j.foodres.2019.05.011

36. Mishra LK, Sangma D. Quality attributes, phytochemical profile and storage stability studies of functional ready to serve (RTS) drink made from blend of Aloe vera, sweet lime, amla and ginger. J Food Sci Technol. 2017; 54(3): 761–9. https://doi.org/10.1007/s13197-017-2516-9

37. Feitosa BF, de Oliveira ENA, de Freitas FBF, de Oliveira Neto JO, Feitosa RM, Lima TLS, Medeiros ML da S, Feitoza JVF, Coutinho HDM. Beverage composed of fruits and soy: Microbiology, colorimetry and effects of refrigerated storage on physical-chemical parameters. Food Biosci. 2022; 49(April). https://doi.org/10.1016/j.fbio.2022.101863

38. Palka A, Wilczyńska A. Storage Quality Changes in Craft and Industrial Blueberry, Strawberry, Raspberry and Passion Fruit-Mango Sorbets. Foods. 2023; 12(14). https://doi.org/10.3390/foods12142733

39. Beckles DM. Factors affecting the postharvest soluble solids and sugar content of tomato (*Solanum lycopersicum* L.) fruit. Postharvest Biol Technol. 2012; 63(1): 129–40. https://doi.org/10.1016/j.postharvbio.2011.05.016

40. Li JL, Sun DW, Cheng JH. Recent Advances in Nondestructive Analytical Techniques for Determining the Total Soluble Solids in Fruits: A Review. Compr Rev Food Sci Food Saf. 2016; 15(5): 897–911. https://doi.org/10.1111/1541-4337.12217

41. Yusof N, Jaswir I, Jamal P, Jami MS. Texture Profile Analysis (TPA) of the jelly dessert prepared from halal gelatin extracted using High Pressure Processing (HPP). Malays J Fundam Appl Sci. 2019; 15(4): 604–8. https://doi.org/10.11113/mjfas.v15n4.1583

42. Morton RD. Aerobic plate count. In: Downes FP, K ITO, editors. Compendium of Methods for the Microbiological Examination of Foods. 4th ed. American Public Health Association; 2001. Chapter 7, p.63-67.

43. Brazil. Ministério da Saúde/Agência Nacional de Vigilância Sanitária. Instrução Normativa nº 161, de 1 de julho de 2022. Estabelece os padrões microbiológicos dos alimentos. ANVISA. Published on Jul 6, 2022.

44. Riesute R, Salomskiene J, Moreno DS, Gustiene S. Effect of yeasts on food quality and safety and possibilities of their inhibition. Trends Food Sci Technol. 2021; 108(September 2020): 1–10. https://doi.org/10.1016/j.tifs.2020.11.022

45. Terezan AP, Junqueira JGM, Wakui VG, Kato L, Oliveira CMA, Martins CHG, Santiago MB, Severino VGP. Qualitative analysis of the acetogenins from *Annona coriacea* (*Annonaceae*) leaves by HPLC-Q-Orbitrap and their antibacterial potential against oral pathogens. Nat Prod Res. 2022; 36(3): 765–71. https://doi.org/10.1080/14786419.2020.180331246.

46. Da Silva J, Cerdeira CD, Chavasco JM, Cintra ABP, da Silva CBP, de Mendonça AN, Ishikawa T, Boriollo MFG, Chavasco JK. Triagem in vitro da atividade antibacteriana de *Bidens pilosa* Linné e *Annona crassiflora* mart. contra *Staphylococcus aureus* resistente à oxacilina (ORSA) provenientes do ambiente aéreo na clínica odontológica. Rev Inst Med Trop Sao Paulo. 2014; 56(4): 333–40. https://doi.org/10.1590/S0036-46652014000400011

47. Ramos ALCC, Silva MR, Mendonça H de OP, Mazzinghy AC do C, Silva VDM, Botelho BG, Augusti R, Ferreira RM de SB, Sousa IMN de, Batista-Santos P, Araújo RLB de, Melo JOF. Use of pulp, peel, and seed of *Annona crassiflora* Mart. in elaborating extracts for fingerprint analysis using paper spray mass spectrometry. Food Res Int. 2022; 160(May). https://doi.org/10.1016/j.foodres.2022.111687

48. Stafussa AP, Maciel GM, Bortolini DG, Maroldi WV, Ribeiro VR, Fachi MM, Pontarolo R, Bach F, Pedro AC, Haminiuk CWI. Bioactivity and bioaccessibility of phenolic compounds from Brazilian fruit purees. Future Foods. 2021; 4(May): 100066. https://doi.org/10.1016/j.fufo.2021.100066

49. Arruda HS, Angolini CFF, Eberlin MN, Pastore GM, Marostica Junior MR. UHPLC-ESI-QTOF-MS/MS Profiling of Phytochemicals from Araticum Fruit (*Annona crassiflora* Mart.) and Its Antioxidant Activity. Foods. 2023; 12(18). https://doi.org/10.3390/foods12183456

50. Hossen MM, Uddin MN, Islam Khan MS, Islam SMH, Hasanuzzaman M, Bithi UH, Abu Tareq M, Hassan MN, Sayeed A, Robbani RB, Mitra K. Nutritional and in vitro antioxidant activity analyses of formulated soymilk dessert. Heliyon. 2022; 8(10). https://doi.org/10.1016/j.heliyon.2022.e11267

51. Vasco C, Ruales J, Kamal-eldin A. Total phenolic compounds and antioxidant capacities of major fruits from Ecuador. Food Chem. 2008; 111(4): 816-823. https://doi.org/10.1016/j.foodchem.2008.04.054

52. Caleja C, Ribeiro A, Barreiro MF, Ferreira ICFR. Phenolic Compounds as Nutraceuticals or Functional Food Ingredients. Curr Pharm Des. 2017; 23(19): 2787–806. https://doi.org/10.2174/1381612822666161227153906

53. Guan H, Zhang W, Sun-Waterhouse D, Jiang Y, Li F, Waterhouse GIN, Li D. Phenolic-protein interactions in foods and post ingestion: Switches empowering health outcomes. Trends Food Sci Technol. 2021; 118(PA): 71–86. https://doi.org/10.1016/j.tifs.2021.08.033

54. Gopika Jayaprakash, Chawla P, Sridhar K, Bains A. Interactions of legume phenols-rice protein concentrate towards improving vegan food quality: Development of a protein-phenols enriched fruit smoothie. Food Res Int. 2023; 171(June): 113075. https://doi.org/10.1016/j.foodres.2023.113075

55. Türkyılmaz M, Hamzaoğlu F, Ünal H, Özkan M. Influence of amino acid addition on the thermal stability of anthocyanins in pomegranate (*Punica granatum* L., cv. Hicaznar) and orange (*Citrus sinensis* L. Osbeck, cv. Valencia) juice blend. Food Chem. 2022; 370(September 2021). https://doi.org/10.1016/j.foodchem.2021.131061

56. Begum N, Khan QU, Liu LG, Li W, Liu D, Haq IU. Nutritional composition, health benefits and bio-active compounds of chickpea (*Cicer arietinum* L.). Front Nutr. 2023; 10(September). https://doi.org/10.3389/fnut.2023.1218468

57. Ozdal T, Capanoglu E, Altay F. A review on protein-phenolic interactions and associated changes. Food Res Int. 2013; 51(2): 954–970. https://doi.org/10.1016/j.foodres.2013.02.009

58. Karadag A, Ozcelik B, Saner S. Review of methods to determine antioxidant capacities. Food Anal Methods. 2008;2(1):41–60. https://doi.org/10.1007/s12161-008-9067-7

59. Thaipong K, Boonprakob U, Crosby K, Cisneros-Zevallos L, Byrne DH. Comparison of ABTS, DPPH, FRAP, and ORAC assays for estimating antioxidant activity from guava fruit extracts. J Food Compos Anal. 2006; 19(6–7): 669-675. https://doi.org/10.1016/j.jfca.2006.01.003

60. Sindhi V, Gupta V, Sharma K, Bhatnagar S, Kumari R, Dhaka N. Potential applications of antioxidants – A review. J Pharm Res. 2013; 7(9): 828–835. https://doi.org/10.1016/j.jopr.2013.10.001

61. Li JM, Nie SP. The functional and nutritional aspects of hydrocolloids in foods. Food Hydrocolloids. 2016; 53 :46–61. https://doi.org/10.1016/j.foodhyd.2015.01.035

62. BeMiller JN. Hydrocolloids. In: Gluten-free cereal products and beverages. Academic Press; 2008. p. 203-215. https://doi.org/10.1016/B978-0-12-373739-7.50011-3

63. Damiani C, Silva EP, Becker FS, Endrigo DE, Asquieri ER, Silva FA da, Boas EV de BV. Antioxidant Potential of Marolo Jam (*Annona crassiflora* Mart) during Storage. OALib. 2017; 04(05): 1–12. https://doi.org/10.4236/oalib.1103158

64. Manzoor M, Singh J, Bandral JD, Gani A, Shams R. Food hydrocolloids: Functional, nutraceutical and novel applications for delivery of bioactive compounds. Int J Biol Macromol. 2020;165:554–567. https://doi.org/10.1016/j.ijbiomac.2020.09.182

65. Boye JI, Aksay S, Roufik S, Ribéreau S, Mondor M, Farnworth E, Rajamohamed SH. Comparison of the functional properties of pea, chickpea and lentil protein concentrates processed using ultrafiltration and isoelectric precipitation techniques. Food Res Int. 2010; 43(2): 537–546. https://doi.org/10.1016/j.foodres.2009.07.021

66. Cohen K de O, Sano SM, Sousa-Silva JC, Silva JT. Avaliação da características físicas e físico-químicas dos frutos de araticum procedentes de Cabeceiras, GO. Bol Pesq Des Embrapa Cerrados. 2010; 270: 16. http://bbeletronica.cpac.embrapa.br/2010/bolpd/bolpd_270.pdf

67. De Medeiros AC, Bolini HMA. Plant-based frozen desserts: Temporal sensory profile and preference. Braz J Food Technol. 2021; 24: 1–12. https://doi.org/10.1590/1981-6723.03720

68. Gorman M, Moss R, McSweeney MB. Sensory perception of ice cream and plant-based alternatives evaluated blinded and with ingredient lists. Food Hum. 2023; 1(September): 1267–1273. https://doi.org/10.1016/j.foohum.2023.09.028

69. Greis M, Nolden AA, Kinchla AJ, Puputti S, Seppä L, Sandell M. What if plant-based yogurts were like dairy yogurts? Texture perception and liking of plant-based yogurts among US and Finnish consumers. Food Qual Prefer. 2023; 107(March). https://doi.org/10.1016/j.foodqual.2023.104848

Figure 1 Click here to access/download;Figure;Figure 1.docx

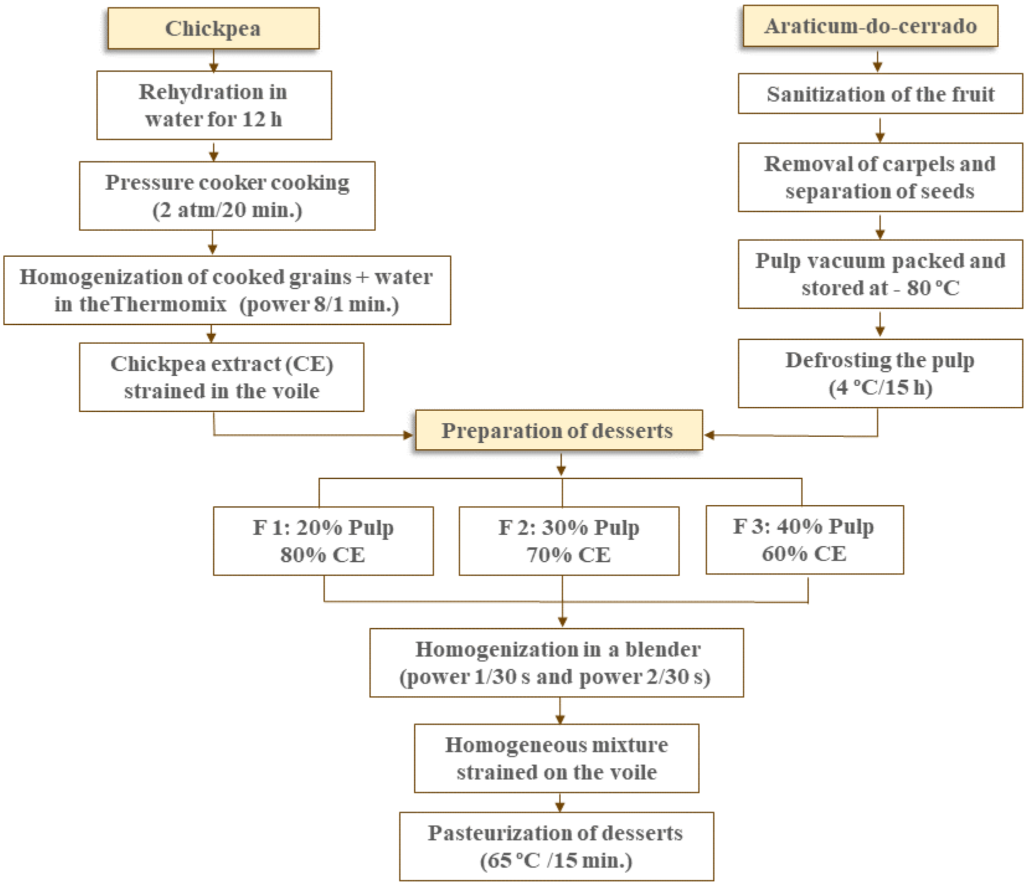

Figure 1. Production Flowchart of the formulations containing different proportions of araticum pulp and chickpea extract.

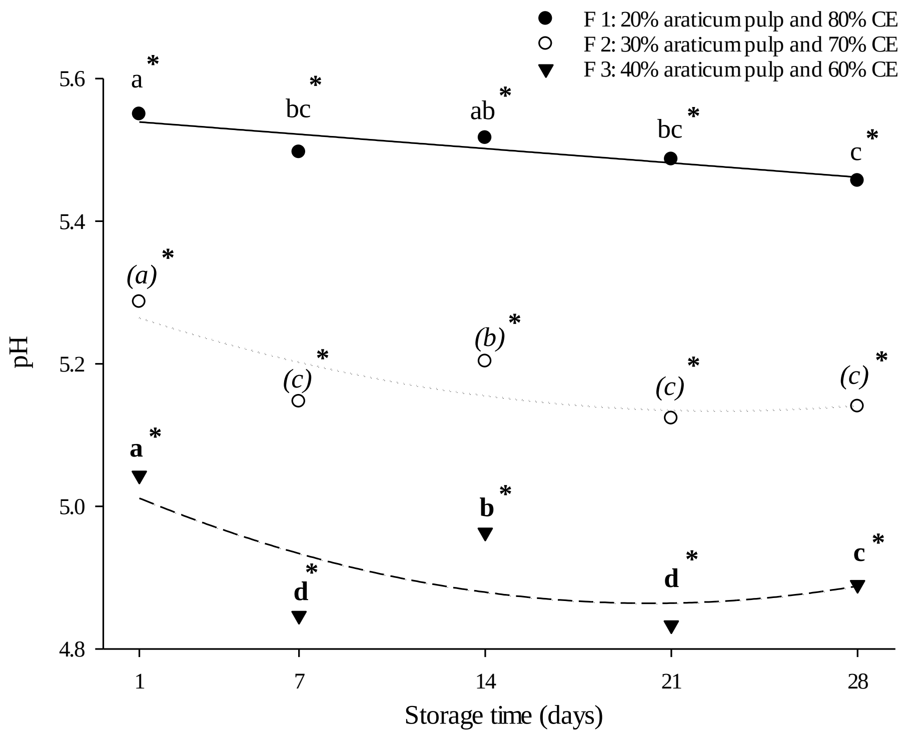

Figure 2. pH regression curves of formulations F1, F2, and F3 during 28 days of storage at 4 ºC. CE = chickpea extract. *Statistical differences comparing the formulations in the same storage time. Different letters within the same formulation indicate statistical differences throughout storage time ($P < 0.05$).

A)

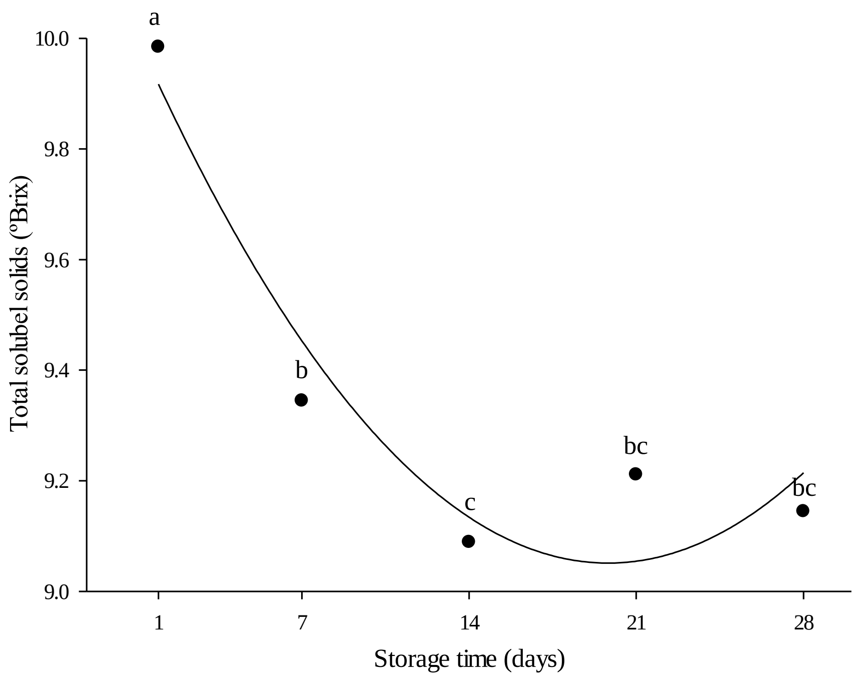

B)

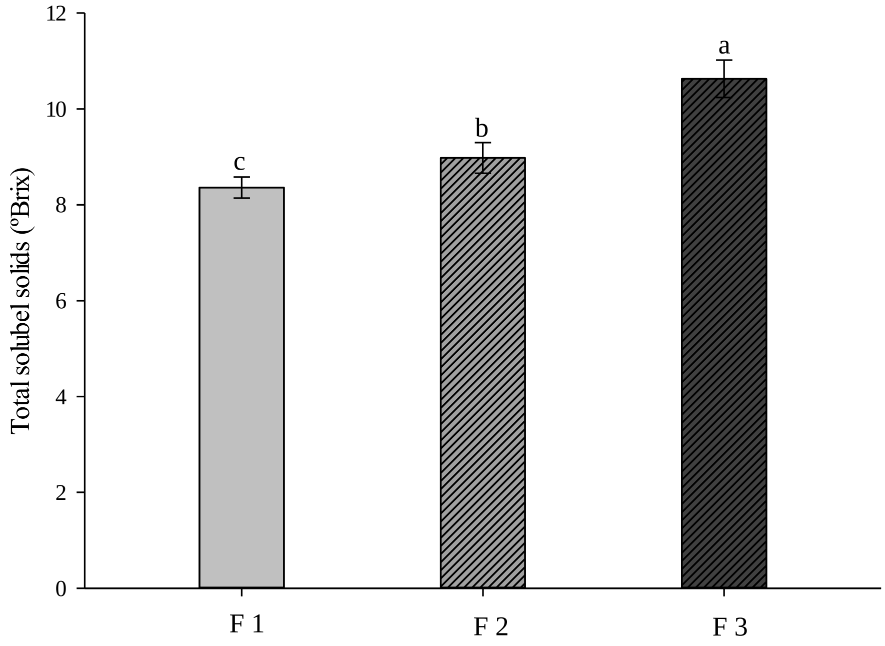

Figure 3. (A) Total soluble solids (ºBrix) values during storage time, independent of the proportion of araticum pulp and chickpea extract used in the formulations. (B) Total soluble solids (ºBrix) values of different formulations independent of the storage time.

F1= 20% araticum pulp + 80% chickpea extract, F2= 30% araticum pulp + 70% chickpea extract, F3= 40% araticum pulp + 60% chickpea extract. Different letters indicate statistical differences (P < 0.05).

Click here to access/download;Figure;Figure 4.docx

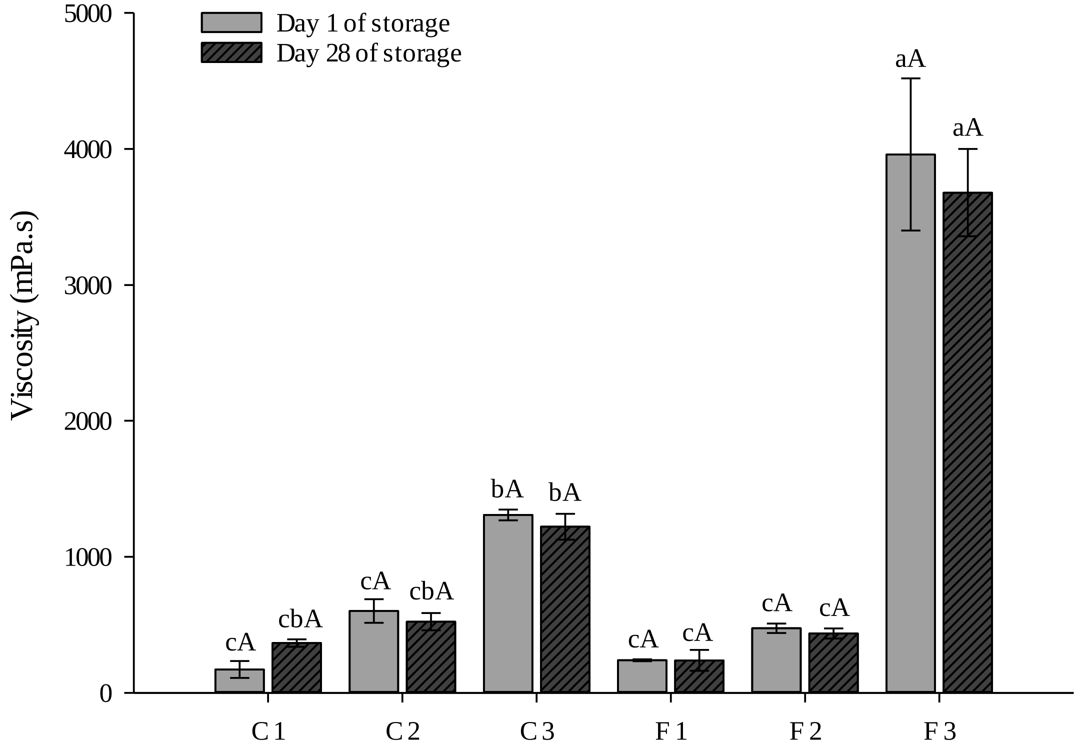

Figure 4. Viscosity (mPa-s) of controls and test formulations on days 1 and 28 of storage at 4 ºC. C 1: 20% araticum pulp + 80% water, C 2: 30% araticum pulp + 70% water, C 3: 40% araticum pulp + 60% water, F 1: 20% araticum pulp + 80% chickpea extract, F2: 30% araticum pulp + 70% chickpea extract, F 3: 40% araticum pulp + 60% chickpea extract. Different lowercase letters indicate statistical differences between formulations and controls; different capital letters indicate differences in the storage time within the same formulation by the Tukey test ($P < 0.05$).

Click here to access/download
Supporting Information
Declaration_responsible_researcher_original_1.pdf

Click here to access/download
Supporting Information
Declaration_responsible_researcher_translated_1.docx

Click here to access/download
Supporting Information
Declaration_responsible_researcher_translated_2.pdf

Click here to access/download
Supporting Information
Declaration_responsible_researcher_original_2.pdf

Responses to questions

Click here to access/download

Supporting Information

Responses_questions.pdf
